# Guided Biofilm Therapy for Management of “Desquamative Gingivitis”—Clinical Cases

**DOI:** 10.3390/clinpract14050153

**Published:** 2024-09-20

**Authors:** Blagovesta Yaneva, Maria Mutafchieva, Petar Shentov, Georgi Tomov

**Affiliations:** 1 Department of Periodontology and Oral Mucosa Diseases, Faculty of Dental Medicine, Medical University of Plovdiv, 4000 Plovdiv, Bulgaria; mariya.mutafchieva@mu-plovdiv.bg (M.M.); petar.shentov@mu-plovdiv.bg (P.S.); 2 Department of Health Care and Social Work, New Bulgarian University, 1618 Sofia, Bulgaria; dr.g.tomov@gmail.com

**Keywords:** desquamative gingivitis, oral lichen planus, pemphigoid, pemphigus, periodontal treatment, guided biofilm therapy, air abrasion

## Abstract

**Background:** Desquamative gingivitis is a clinical manifestation often associated with various mucocutaneous disorders, characterized by red, painful, and friable gingiva. It is predominantly seen in middle-aged to elderly females and is typically linked to autoimmune conditions such as lichen planus, pemphigoid, and pemphigus, among others. Due to the chronic pain and difficulty in maintaining personal oral hygiene, professional care becomes crucial. **Methods:** This article explores the application of guided biofilm therapy as a novel, gentle approach for managing desquamative gingivitis, focusing on three clinical cases. This therapy employs erythritol-based powders for biofilm removal, offering a less abrasive and more comfortable alternative to traditional mechanical plaque removal techniques. **Results:** The cases demonstrate the effectiveness of guided biofilm therapy in reducing discomfort and improving clinical outcomes in desquamative gingivitis patients, particularly those suffering from mucous membrane pemphigoid, pemphigus vulgaris, and oral lichen planus. **Conclusions:** The guided biofilm approach underscores the importance of tailored periodontal therapy in managing nonplaque-induced gingival lesions, improving patient compliance and oral health outcomes.

## 1. Introduction

Desquamative gingivitis (DG) is a clinical term used to describe the local manifestation of some mucocutaneous conditions on the gingiva. It is characterized by red, painful, glazed, and friable gingiva. In the majority of cases, DG is caused by autoimmune disorders, in particular lichen planus, pemphigoid, and pemphigus. However, other conditions like chronic ulcerative stomatitis, erythema multiforme, lupus erythematosus, psoriasis, linear IgA disease, Crohn’s disease, allergic reactions to toothpaste/mouth rinses (plasma cell gingivitis), etc., may also present clinically as desquamative gingivitis [1]. DG is not a diagnosis per se. The last classification scheme for periodontal and peri-implant diseases and conditions categorizes desquamative gingivitis under the non-plaque induced gingival lesion caused by inflammatory and immune conditions [2]. It is extremely important for the clinician to be able to differentiate desquamative gingivitis from classic plaque-induced gingivitis. Desquamative gingivitis is not caused by inflammation and does not demonstrate some of the typical signs of gingivitis like swelling or bleeding. The red appearance of the gingiva in DG is due to atrophy of the epithelium, with or without concomitant desquamation or erosion. Additionally, the alterations usually involve the attached gingiva but spare the free gingiva (gingival margin and interdental papillae). Plaque-induced gingivitis peaks around puberty, while desquamative gingivitis is usually seen in middle-aged to elderly females. Most importantly, in contrast with plaque-induced gingivitis, DG is extremely painful. Patients avoid flossing and brushing, which results in the deposition of plaque and calculus and leads to secondary plaque-induced gingivitis with superimposition of symptoms [3]. Thus, professional oral hygiene in patients with DG is extremely important and could contribute to the improvement of the disease [4].

MMP is an autoimmune blistering disease, predominantly affecting mucous membranes, including the oral, laryngeal, oesophageal, and ocular mucous membranes. Skin is very rarely involved. Antibodies are produced against certain target antigens found in the basement membrane, resulting in the epithelium being detached from the underlying lamina propria. The clinical manifestation of these histological changes is sub-epithelial blister formation [5]. Elderly women are most frequently affected. Intra-orally, the most common presentation of MMP is desquamative gingivitis. In addition to the clinical appearance described above, areas of erosions, partially covered by the ruptured blister’s roof, might also be observed on the gingiva [6]. Occasionally, other sites like the buccal mucosa, palate, and tongue could be affected, presenting with blood-filled bullae or fibrin-covered irregular erosions. The chief complaint of gingival soreness makes patients repeatedly seek dental help until a correct diagnosis is made. Histological examination and direct immunofluorescence to detect linear deposits of IgG and C3 at the basement membrane zone are needed to confirm the diagnosis of MMP. Then, consultation with an ophthalmologist is highly recommended, even in the absence of eye complaints.

The oral lesions of MMP have a chronic course and are usually recalcitrant. As with most autoimmune diseases, the first choice of treatment is topical corticosteroids. In cases with desquamative gingivitis, medication is applied in a custom-made tray to elongate the time of contact with the tissue. Plaque control and regular professional cleaning improve the condition [1].

Pemphigus vulgaris is another autoimmune disease, characterized by blistering of the skin and mucous membrane [6]. It is a serious, potentially lethal condition, in which antibodies are directed against the epithelial desmosomes and cause acantholysis, resulting in blister formation. The cause of this immune aggression is still unknown, but factors like viruses, drugs, etc., are assumed to be involved. Middle-aged females are predominantly affected. Oral manifestation is present in half of the patients and often precedes the skin lesions. Loose bullae with clear serous content are typical of PV. However, in the mouth, the blebs are of very short duration. Formed in the epithelium thickness, the blisters are thin and rapture almost immediately, leaving painful bright-red erosions. The lesions are multiple and can be found anywhere in the oral cavity. 

Desquamative gingivitis manifests as its classic appearance of a fiery red gingiva, but loose blebs or eroded areas may also be noticed. Gentle pressure on the gingiva results in the separation of the epithelium (positive Nikolsky sign). Normal activities, such as food and beverage consumption and maintaining oral hygiene, are extremely painful for the patient [5]. Timely diagnosis of PV is important since it is a serious, potentially rapid progressive disease. Separation of the epithelial cells with cleft formation in the spinous cell layer, acantholytic Tzanck cells, and mild inflammatory infiltrate are among the typical histological findings of the disease. Direct immunofluorescence is, however, a gold standard in the diagnosis, demonstrating intercellular deposits of IgG and C3 in a specific fish-net pattern. Indirect immunofluorescence in PV is also positive, and usually the antibodies titre corresponds to the severity of the disease [1]. Systemic corticosteroids are indicated to prevent further spread and worsening of the disease.

Lichen planus is a relatively common mucocutaneous disease that frequently affects oral mucosa. Despite having an unknown aetiology, emotional stress and anxiety are among the mentioned triggers of the disease. Regarding pathogenesis, OLP is an immunologically mediated condition in which auto-cytotoxic CD8+ T-Ly destroy the basal cells of the oral epithelium. Women in their fifth and sixth decades of life are most commonly affected. In the oral cavity, lichen planus may present in six different clinical forms: reticular, papular, plaque-like, atrophic, bullous, and erosive. However, white keratotic lines in a lace-like pattern (Wickham striae) are a pathognomonic sign of the disease. Other characteristic features are multi-site involvement and bilateral, symmetric distribution of the lesions. Sites of predilection are the buccal mucosa, followed by the tongue and the gingiva. OLP is characterized by a chronic course with alternating periods of exacerbation and remission. In 2005, the WHO classified OLP as an oral potentially malignant disorder (OPMD). Based on the clinical and scientific evidence that had been enriched over nearly twenty years of research, the malignant potential of the disease was confirmed again in the latest report on the nomenclature and classification of OPMDs in 2021 [6]. Histological examination to reveal a subepithelial, dense, band-like infiltrate of lymphocytes with basal cell liquefactive (hydropic) degeneration is needed to confirm the diagnosis of OLP and exclude dysplasia.

Approximately 10% of patients with oral lichen planus have lesions confined only to the gingiva. These may occur as some of the following distinctive patterns: white keratotic lesions in the form of lines, papules, or more homogenous plaque; areas of erosion and possibly white striae at the periphery; and, rarely, vesicles or bullae. Only the atrophic form of OLP manifests clinically as classic desquamative gingivitis with diffuse erythema as a result of epithelial thinning. Patients complain of persistent soreness of the gums, which worsens with spicy foods and when maintaining oral hygiene.

Conventional professional mechanical plaque removal using ultrasonic devices in combination with polishing brushes and paste may cause a lot of discomfort to patients with DG due to accidentally touching the lesions. Therefore, much more comfortable treatment modalities are needed. Guided biofilm therapy (GBT) is a new treatment approach aiming for more gentle professional mechanical plaque and calculus removal. Plaque disclosing before professional oral hygiene facilitates later treatment and helps motivate the patient to adopt a better personal oral hygiene regimen [5]. The powder used for GBT consists of erythritol, which is a white, crystalline powder with a sweet taste and much smaller particles (14 µm) in comparison to the most widely used air polishing powder—sodium bicarbonate (40–200 µm). Erythritol is a less-abrasive polishing powder that provides safe enamel, dentin, and cementum polishing, and is used for subgingival polishing [7,8,9]. Another low abrasive polishing power with comparable effectiveness is the glycine polishing powder. It has been tested on gingival tissue, demonstrating less invasiveness and tissue trauma compared to sodium bicarbonate [10,11,12]. Although there are no studies demonstrating the action of erythritol on gingival tissues, we suppose it would be gentler in comparison to conventional polishing with brush and paste or polishing with sodium bicarbonate. Moreover, the use of erythritol has been reported to provide more comfortable instrumentation for patients during initial and supportive periodontal therapy [8,13]. 

## 2. Materials and Methods

**Figure 1:**
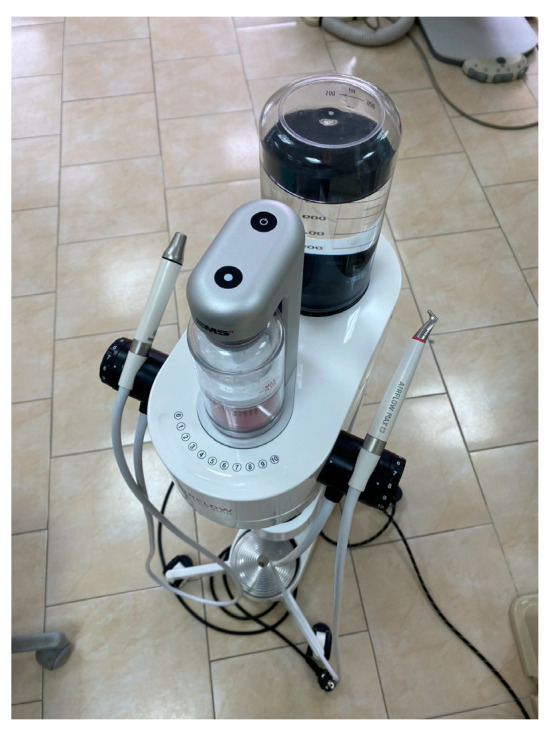
Combined ultrasonic and polishing device.

The following article presents 3 clinical cases with desquamative gingivitis with different aetiology treated by means of guided biofilm therapy. All the patients were treated with the following protocol. Biofilm disclosing was performed with Biofilm discloser^®^ (EMS, Nyon, Switzerland) to demonstrate the biofilm in two colours—reddish for the new plaque formation and blue for mature biofilm. Professional mechanical plaque removal was performed with AIRFLOW^®^ Prophylaxis Master (EMS, Nyon, Switzerland) in two steps (Figure 1). First, the disclosed biofilm was removed with erythritol-based EMS AIRFLOW^®^ PLUS powder. After that, the supra- and subgingival calculus deposits were removed with a PIEZON^®^ ultrasonic scaler handpiece with a PS instrument and a low frequency of 25 kHz. The treatment is performed with a regulated irrigation temperature of 40 °C and activated by a wireless foot pedal. After completion of the instrumentation patients’ tolerance to the procedure was assessed using a visual analogue scale (VAS), where 0 was no pain and discomfort at all and 10 represented the greatest possible discomfort during instrumentation [14].

### 2.1. Clinical Case 1—Mucous Membrane Pemphigoid (MMP)

**Figure 2:**
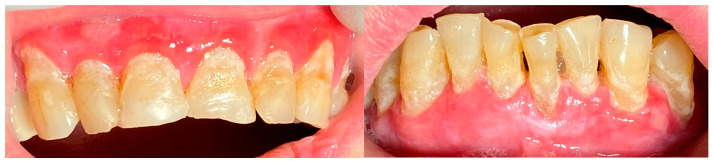
Patient with mucous membrane pemphigoid.

**Figure 3:**
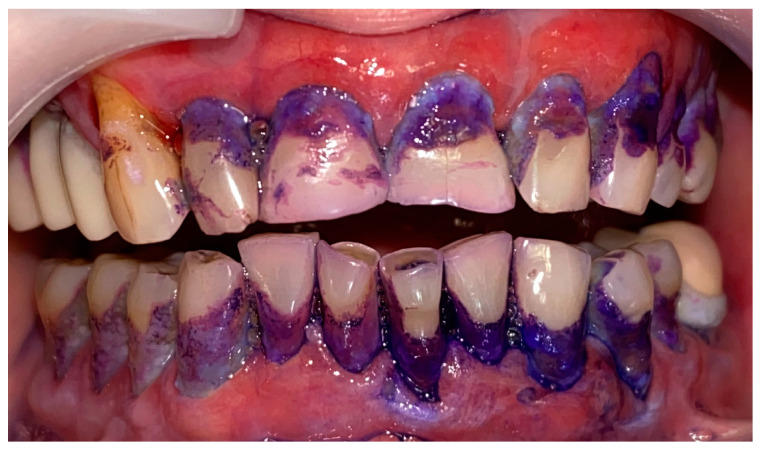
Patient with mucous membrane pemphigoid. Disclosing of the biofilm.

**Figure 4:**
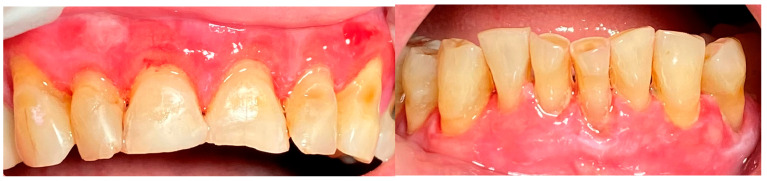
Patient with mucous membrane pemphigoid. Immediately after GBT.

A 72-year-old female patient with a long history of MMP (Figure 2) came to our clinic for routine mechanical periodontal instrumentation. The diagnosis of MMP with desquamative gingivitis was made three years ago. Since then, the patient has had her teeth professionally cleaned every 6 months. According to the patient, the procedure was always related to severe pain and often provoked exacerbation of the disease with new lesion formation. Upon disclosing the biofilm, a 100% full mouth plaque score (FMPS = full mouth % of sites with plaque) was recorded (Figure 3). The full mouth bleeding (FMBS) score was not measured due to the severe pain from the gingiva. She then underwent the GBT protocol, including teeth polishing with erythritol-based powder and ultrasonic instrumentation. The therapy was effective (Figure 4) and caused minimal discomfort, with a score of 1 according to the visual analogue scale (VAS) used. The patient was invited for a follow-up examination after three days. No new lesions were detected.

### 2.2. Clinical Case 2—Pemphigus Vulgaris (PV)

**Figure 5:**
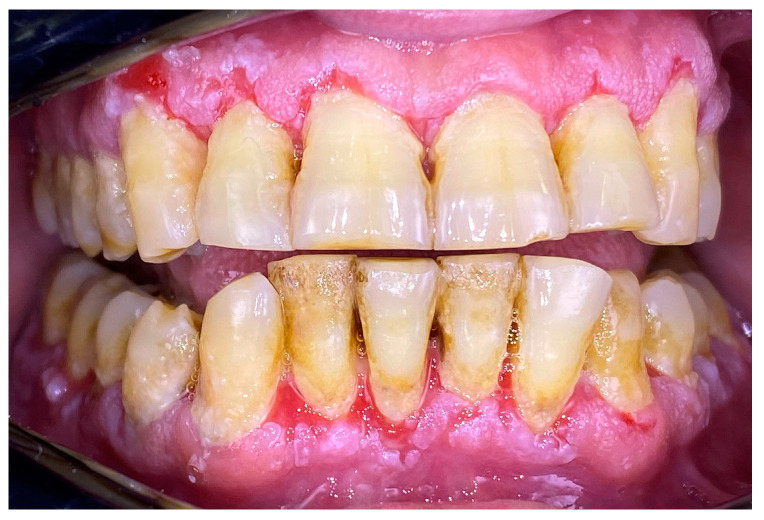
Patient with pemphigus vulgaris.

**Figure 6:**
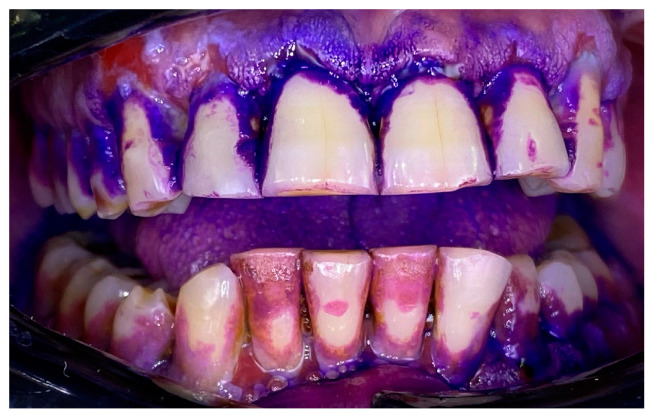
Patient with pemphigus vulgaris. Disclosing of the biofilm.

**Figure 7:**
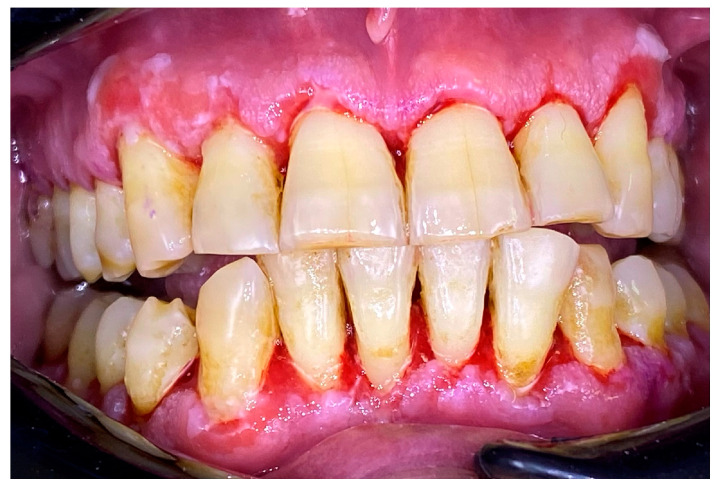
Patient with pemphigus vulgaris. Immediately after GBT.

A 67-year-old male patient came to our clinic with chief complaints of severe oral pain that interferes with food and beverage intake, bad breath, and difficult personal oral hygiene. The patient presented with irregular bright red erosions on the gingiva as well as the hard and soft palate. Light pressure on the gingiva resulted in detachment of the epithelium (Figure 5). The results of the applied histological examination and direct immunofluorescence confirmed the diagnosis of PV. The patient was referred to a dermatologist who took over the management of the disease. However, after consultation with the primary care provider, professional mechanical plaque removal was planned. The FMPS revealed 100% because of the difficulty in performing personal oral hygiene. The FMBS was not tested because of severe pain from the gingiva. We used GBT with the abovementioned protocol (Figure 6). The therapy was effective in biofilm control (Figure 7) and the patient reported much more comfort during the procedure in comparison to the conventional one he had before (VAS—0).

### 2.3. Clinical Case 3—Oral Lichen Planus (OLP)

**Figure 8:**
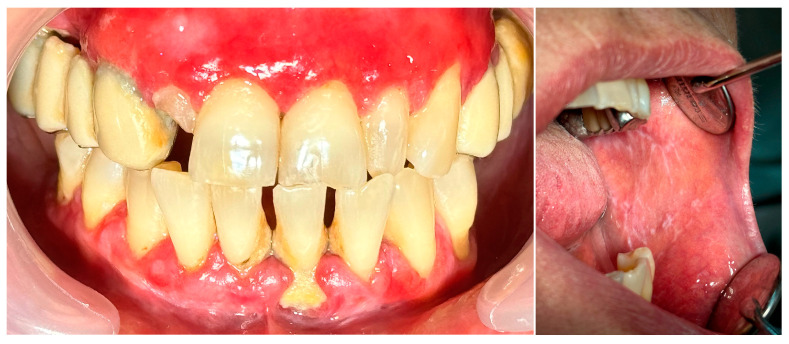
Patient with oral lichen planus.

**Figure 9:**
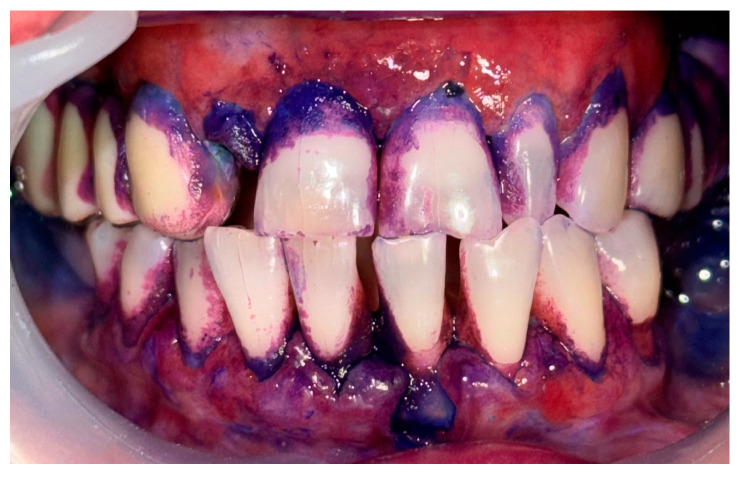
Patient with oral lichen planus. Biofilm disclosure and immediately after GBT.

**Figure 10:**
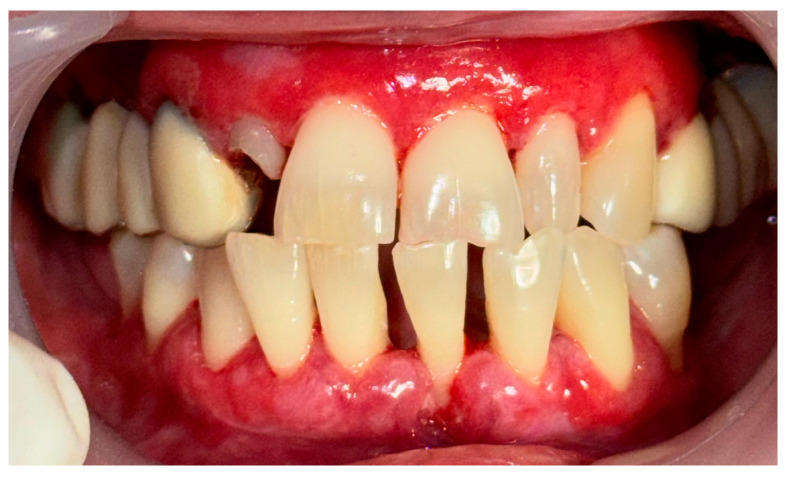
Patient with oral lichen planus. Biofilm disclosure and immediately after GBT.

A 56-year-old woman came to the Department of Periodontology and Oral Mucosa Diseases with complaints of gums soreness and inability to maintain proper oral hygiene. The clinical examination revealed extensive red atrophic fields on the attached gingiva of the maxilla and mandible. Excessive amounts of plaque and calculus, accompanied by swollen interdental papillae, were also part of the clinical findings. In addition, slightly elevated white striations were found on the buccal mucosa bilaterally (Wickham striae) (Figure 8). Given that these are considered a hallmark of the disease, the patient was clinically diagnosed with oral lichen planus (OLP). Interestingly, the patient had not noticed these mucosal lesions until then. This once again demonstrated the importance of a routine annual examination by an oral pathology specialist to prevent oral cancer, given that OLP is a precancerous condition. An incisional biopsy was then performed with the Er:YAG laser using the following parameters: pulse mode, 35 Hz, 7 W, 200 mJ, and the diagnosis was confirmed histologically. As a first step of treatment, 0.05% Clobetasol propionate tissue adhesive gel was prescribed for one week, aimed at mild relief of patient symptoms to cope with the following procedures—periodontal instrumentation and taking impressions for custom-made trays for targeted corticosteroid treatment. Plaque and calculus removal was performed using GBT with the abovementioned protocol (Figure 9). The procedure did not provoke extreme bleeding or micro-erosions on the gingiva and the patient reported a score of 0 in the VAS (Figure 10). Custom-made trays were then fabricated to further target corticosteroid treatment.

A summary of all patients’ medical data records is presented in Table 1.

## 3. Discussion

Desquamative gingivitis is a particular/specific non-plaque-induced periodontal disease that often requires specialist cooperation for its treatment. The purpose of therapy is to decrease/cease the disease progression and improve the patient’s quality of life. Since autoimmunity is implicated in the aetiology of DG, corticosteroids are considered a gold standard for its treatment. Priority should be given to topical agents. However, in severe cases, systemic medications are indicated [3].

It is important to note that the immunological reaction occurring in mucocutaneous disorders itself does not result in clinical attachment loss and periodontitis [1]. However, the symptoms associated with such lesions hamper proper oral hygiene maintenance by patients and thus may predispose them to the occurrence of long-term periodontal disease. The bacterial biofilm formation and calculus deposits initiate gingival inflammation. However, the aetiology of periodontitis is much more complex and multifactorial, influenced by dysbiotic ecological changes within the microbiome, immune-inflammatory host response type, genetic factors, systemic diseases, smoking, etc. A systematic review and meta-analysis conducted by Sanadi R.M. et al. analysed the association between periodontal disease and oral lichen planus. They found that among the studied periodontal parameters, bleeding on probing and probing depth were significantly associated with oral lichen planus [15,16]. On the other hand, biofilm accumulation leading to secondary gingival inflammation may further exacerbate the lichenoid lesion. In 2021, another systematic review tested the effect of plaque control on OLP with gingival manifestations and concluded that oral hygiene intervention was associated with improvements in clinical disease status (Escudier index) and patient-reported outcomes where the Oral Health Impact Profile-14 (OHIP-14) has been used to assess the impact that oral health problems can have on an individual’s life [17]. All this underlines the importance of professional debridement as a method which not only prevents further periodontal destruction but also improves OLP clinical scores and the quality of life of patients. Unlike plaque-induced gingivitis, desquamative gingivitis is related to moderate to severe pain. What is more, typical of lichen planus is the so-called Koebner phenomenon—the appearance of new skin lesions on previously unaffected skin secondary to trauma [18]. Thus, it could be speculated that mechanical trauma during instrumentation will cause not only pain but may also lead to exacerbation and the formation of new lesions. Based on the above, it is extremely important for the clinician to select the most painless and atraumatic method possible for professional debridement.

Periodontal maintenance is an integral part of the treatment of patients with DG. It should be minimally invasive without causing further complications to the oral mucosa. Regular visits are required. Air abrasive systems have been demonstrated to remove dental biofilm effectively. However, the application of high-pressure water settings may cause additional discomfort in patients with DG [19]. The erythritol-based powder has smaller particles and seems to demonstrate a better acceptance rate from the patients [20]. Therefore, GBT, representing plaque removal guided by disclosing agents using gentle powders with warm water, could significantly improve patients’ perception and dentists’ operations.

## 4. Conclusions

In conclusion, the periodontal management of desquamative gingivitis through guided biofilm therapy (GBT) highlights the effectiveness of this approach in treating complex gingival conditions. Clinical improvement and symptomatic relief emphasize the importance of meticulous plaque control and targeted biofilm disruption in managing gingival diseases associated with mucocutaneous disorders. Further studies with larger cohorts are recommended to substantiate these findings and establish standardized protocols for GBT in the treatment of desquamative gingivitis.

## Figures and Tables

**Table 1:** Patients’ data summary.

	Case 1	Case 2	Case 3
**Diagnosis**	Mucous membrane pemphigoid (MMP)	Pemphigus vulgaris (PV)	Oral lichen planus (OLP)
**Gender**	Female	Male	Female
**Age**	74	67	56
**Oral lesions medication**	0.05% Clobetasol propionate tissue adhesive gel Prednisolone 5 mg/day Azathiorpine 50 mg/day	0.05% Clobetasol propionate tissue adhesive gel Prednisolone 5 mg twice/day Azathiorpine 50 mg twice/day	0.05% Clobetasol propionate tissue adhesive gel for 4 weeks
**Other comorbidities**	Essential hypertension One-angle glaucoma (OAG)	No	Past colon cancer (colostomy) Essential hypertension
**Other drugs intake**	Bisoprolol 5 mg/day Latanoprost 50 mcg/mL Eye drops one a day	No	no
**Allergies**	Not reported	Not reported	Not reported

## Data Availability

The data presented in this study are available on request from the corresponding author.
